# Reliability and validity of the Japanese version of the Gratitude at Work Scale (GAWS)

**DOI:** 10.1002/1348-9585.12185

**Published:** 2020-12-20

**Authors:** Yu Komase, Kazuhiro Watanabe, Natsu Sasaki, Norito Kawakami

**Affiliations:** ^1^ Department of Mental Health Graduate School of Medicine The University of Tokyo Japan; ^2^ Japan Society for the Promotion of Science Chiyoda‐ku Japan

**Keywords:** gratitude, psychometric properties, scale development, well‐being, workplace

## Abstract

**Objectives:**

Workplace gratitude is important for improving work‐related outcomes and individual well‐being. Although the gratitude at work scale (GAWS) was developed in the United States, it has not been corroborated in Asian countries with interdependent cultures. This study aimed to develop and validate the GAWS among Japanese workers.

**Method:**

Japanese workers completed online surveys at baseline (N = 206) and 2 weeks later (N = 103). The Japanese GAWS was developed according to the international guidelines. We measured (a) trait gratitude as comparison for the criterion‐related validity, (b) work‐related outcomes/factors (eg, work engagement), and (c) well‐being (eg, eudemonic well‐being at work) as comparisons for convergent validity. Cronbach's alpha, intra‐class correlation coefficients (ICCs), and measurement errors were calculated to assess reliability; measurement validity was evaluated by correlational analyses and confirmatory factor analysis (CFA).

**Results:**

A total of 206 and 93 workers were included for baseline and follow‐up analyses, respectively. Cronbach's alpha and ICCs of the Japanese GAWS ranged from 0.81 to 0.91. CFA showed that the 2‐factor model (ie, gratitude for (a) a supportive work environment and (b) meaningful work) demonstrated a good fit (χ^2^ (34) = 67.58, CFI = 0.967, TLI = 0.956, RMSEA = 0.069, SRMR = 0.037), similar to the original version. As we had hypothesized, overall GAWS and the two domains were significantly correlated with trait gratitude, work‐related outcomes/factors, and well‐being.

**Conclusions:**

The Japanese GAWS demonstrated good reliability and validity. Future research should explore mechanisms related to workplace gratitude and further intervention studies among workers.

## 1 INTRODUCTION

Workplace gratitude is the tendency to recognize and be thankful for how various aspects of a job affect one's life.^1^ According to Cain, gratitude for (a) a supportive work environment and (b) meaningful work are the two components of workplace gratitude.^1^ Moreover, workplace gratitude for a specific object is distinguished from other positive feelings, such as job satisfaction, because the resulting feeling is evoked from experience beneficial to, but not attributable to, the self.^2^ Ferh et al (2017) argued that workplace gratitude is not elicited by an experience itself but by its interpretation.^3^ Therefore, in the same workplace, gratitude levels can vary among individuals, which could be explained by differences in individual attitudes.

Workplace gratitude is important because it improves both work‐related outcomes and individual well‐being.^2^, ^4^ Specifically, previous observational studies in the workplace have reported that gratitude is positively associated with job satisfaction,^5^ relationships and social support,^6^ organizational citizenship behaviors,^7^ and job performance,^8^ and negatively associated with emotional exhaustion or burnout.^5^ Moreover, randomized controlled trials have found positive correlations between gratitude and well‐being. For example, creating a gratitude list effectively improves well‐being, such as positive or negative affect and life satisfaction.^9^, ^10^, ^11^ Collectively, increased worker gratitude is positive for both companies and workers.

To further study its positive effects, workplace gratitude should be measured psychometrically. This can help assess gratitude levels of employees in the workplace or develop new gratitude intervention programs. To date, several measures of workplace gratitude have been developed.^1^ For example, Cameron et al^12^ devised the Positive Practices Survey (PPS) that includes 2 items measuring the expression of gratitude to assess ethical behaviors in the workplace. Although PPS can assess gratitude expressed as a behavior, it cannot compartmentalize personal feelings of workplace gratitude, as mentioned by Cain.^1^, ^12^ Additionally, Lanham et al^4^ developed a 4‐item measure of workplace gratitude, assessing gratitude for a supportive work environment (eg, coworkers and supervisors) but not for meaningful work.^4^ Moreover, psychometric properties have not been examined in either case.^4^

The Gratitude at Work Scale (GAWS) was developed to overcome the aforementioned issues.^1^ Specifically, the creators of GAWS initially conducted a review of gratitude studies to conceptualize workplace gratitude. Subsequently, they identified key work aspects that influence employees’ evaluation of their jobs. Nine items were then added to the Lanham workplace gratitude measure to develop a comprehensive measure of workplace gratitude. Finally, the GAWS creators demonstrated the psychometric properties of the 10‐item GAWS in three studies (3 items that did not meet structure criteria were excluded). GAWS contains two subfactors, gratitude for (a) supportive work environment and (b) meaningful work, and has proven reliable and valid. However, GAWS has not been further developed or validated in samples from countries other than the United States. Testing the effectiveness of GAWS in Asian countries is particularly important since Asian culture is typically more interdependent compared to Western culture, which focuses more on individuality.^13^ We hypothesized that workplace gratitude also plays an important role in Asian populations.

This study examined internal consistency, test‐retest reliability, structural validity, and convergent validity of a translated version of the GAWS among Japanese workers. We hypothesized that the Japanese GAWS would have good internal consistency, test‐retest reliability, and 2‐factor structural validity. Based on the original and relevant previous studies, we developed the following hypotheses to confirm the criterion‐related validity and convergent validity: the Japanese GAWS would correlate significantly with (a) trait gratitude^1^ to support the criterion‐related validity, (b) work‐related outcomes or factors (work engagement, organizational citizenship behavior, work performance, social support from supervisors and colleagues, and workplace social capital),^1^, ^6^, ^7^ and (c) well‐being (positive or negative affect, job satisfaction, life satisfaction, and eudaimonic well‐being)^1^, ^11^ to support the convergent validity. For work‐related outcomes, work engagement was adopted per the original study.^1^ Another study examined this association and confirmed that gratitude is related to work engagement via self‐efficacy.^14^ Although the original study examined the associations of organizational commitment and workplace climate with psychosocial factors at work, we selected social support from supervisors and colleagues and workplace social capital, as these factors are more commonly used in Japan. We also explored the relationship between GAWS on the one hand and job demands and job control on the other. These variables are an important part of the Job Demands‐Resources model, known as the major occupational model, that aims to explain the health impairment and motivational processes.^15^ Concerning the magnitude of the correlations, we assumed equal strength of correlation between the Japanese GAWS and the variables that were also examined in the original study (trait gratitude, work engagement, work performance, psychosocial factors at work, positive or negative affect, job satisfaction, and life satisfaction).^1^ Psychosocial factors at work have been noted to extend to the individual (eg, personality and attitudes).^16^ Although we measured these factors using a different scale than in the original study, we assumed the same correlation strength. The magnitude of correlations between the other variables (organizational citizenship behavior and eudaimonic well‐being) was assumed based on the previous meta‐analyses examining prosociality^17^ and well‐being.^18^ Specifically, we assumed moderate‐to‐large positive correlations (r ≥ 0.30) of (a) trait gratitude, (b) work‐related outcomes or factors, and (c) well‐being (except work performance, life satisfaction, and negative affect) with GAWS,^19^ weak‐to‐moderate positive correlations of work performance and life satisfaction with GAWS (r ≤ 0.30), and negative correlations for negative affect.^1^, ^19^

## 2 METHODS

### 2.1 Design

This study included online surveys at baseline (May 2020) and at 2‐week follow‐up (June 2020) in Japan. The internal consistency, structural validity, and convergent validity of Japanese GAWS were examined using cross‐sectional data. Test‐retest reliability was examined using longitudinal data 2 weeks after the follow‐up. This manuscript follows the COnsensus‐based Standards for the selection of health Measurement INstruments (COSMIN) reporting guidelines.^20^ Each characteristic of the measure was reported according to the COSMIN checklist.

### 2.2 Participants

Participants were selected from among workers who registered as respondents of an Internet‐based survey company, Macromill, Inc^21^ Of the available respondents, 206 workers responded to a web‐based questionnaire. Macromill had obtained a relatively representative sample by accessing >10 million potential participants representing all prefectures in Japan. Participants were recruited based on demographic attributes. Participant inclusion criteria were being (a) full‐time workers living in a prefecture of Japan and (b) aged ≥20 years. There were no exclusion criteria. On the basis of these criteria, the survey company recruited workers from their potential participant pool, until the targeted number was reached (the calculated sample size was 193. See also the Analysis section). Eligible workers who agreed with the terms and conditions of the online survey could access the self‐report questionnaire. Two weeks later, the company randomly sampled 103 participants among the baseline survey respondents. Survey respondents received approximately 50 "Macromill points" for each survey, which could be used for shopping or cashing out (1 point was equivalent to 1 Japanese Yen). Informed consent was obtained from all participants as part of the survey instructions, which explained that any identifying information would be removed from the data and assured protection of personal information. The study protocol was approved by the research ethics committee of the Graduate School of Medicine and the Faculty of Medicine, The University of Tokyo, Japan (No. 2019216NI).

### 2.3 Measurements

Participants were asked to respond twice to the surveys. The surveys included the Japanese GAWS and questions regarding (a) trait gratitude, (b) work‐related outcomes or factors (work engagement, organizational citizenship behavior, work performance, and work psychosocial factors), and (c) well‐being (positive or negative affect, job satisfaction, life satisfaction, and eudaimonic well‐being).

Japanese GAWS was used to measure two factors governing workplace gratitude: (a) supportive work environment (SWE, 6 items) and (b) meaningful work (MW, 4 items). Each GAWS factor score was calculated as an average of the item scores. The overall GAWS score was the average of the 10 items. All items were rated on a 5‐point Likert‐type scale ranging from 1 (never) to 5 (always).

The Japanese GAWS was developed per the procedure specified in the International Society of Pharmacoeconomics and Outcomes Research task force guidelines.^22^ We first obtained permission from the developer of the original GAWS to translate the measures into Japanese. Forward‐translation was independently conducted by YK and NS. We are native Japanese speakers living in Japan and conduct research in occupational positive psychology. We then performed reconciliation, back‐translation, back‐translational review, harmonization, and cognitive debriefing. The back‐translation was conducted by a native English translator unaware of the original scale. The original developer confirmed the back‐translated measures and made revisions at the back‐translation review stage. Cognitive debriefing sessions were conducted with eight Japanese workers recruited using snowball sampling and included a researcher, clinical psychologist, social welfare specialist, physical therapist, local public service worker, white‐collar manufacturing worker, and nurses (n = 2). They were asked to complete the harmonized measure and revise any difficult wording; their feedback was used for further modifications. Results from these stages were combined to develop the final measure. The full version of the Japanese GAWS is provided in Appendix A1.

### 2.4 Trait gratitude

Trait gratitude was measured using the Japanese version of the 5‐item Gratitude Questionnaire (GQ‐5), with items such as "I have so much in life to be thankful for."^23^ The reliability and validity of the Japanese version of the GQ‐5 were confirmed in a previous study.^23^ All items were scored on a 7‐point Likert scale from 1 (strongly disagree) to 7 (strongly agree). The scores of all 5 items were averaged and used for analyses (Cronbach's α = 0.88).

### 2.5 Work engagement

Work engagement was measured using the 9‐item Japanese Utrecht Work Engagement Scale (UWES)^24^ that consists of three subscales: vigor (3 items; eg, "At my job, I feel strong and vigorous"), dedication (3 items, eg, "I am enthusiastic about my job"), and absorption (3 items; eg, "I am immersed in my work"). All items were scored on a 7‐point Likert scale, ranging from 0 (never) to 6 (always). Reliability and unidimensional validity of the Japanese UWES were verified in a previous study.^24^ The overall score (Cronbach's α = 0.95) and three subscales were averaged and used for analyses.

### 2.6 Organizational citizenship behavior

Organizational citizenship behavior was measured using the Japanese Organizational Citizenship Behavior Scale (OCBS).^25^ In Japan, supporting an organization includes voluntary employee work during off‐duty hours. The OCBS consists of 8 items, such as "I assist the supervisor without being asked," and "I deal with visitors who go to other sections of the company." These items were scored on a 5‐point scale, ranging from 1 (not at all) to 5 (very frequently). The reliability and validity of the Japanese OCBS were confirmed previously.^25^ The scores from the 8 items were averaged and used for analyses (Cronbach's α = 0.89).

### 2.7 Work performance

Work performance was measured using 1 item from the validated Japanese short version of the WHO Health and Work Performance Questionnaire (WHO‐HPQ),^26^ which scored an individual's past month's overall job performance on a scale of 0 (worst job performance) to 10 (best). The ratings were multiplied by 10 to calculate work performance per the WHO‐HPQ scoring guidelines.^26^

### 2.8 Psychosocial factors at work

Job demands (3 items; eg, "I have an extremely large amount of work to do"; α = 0.75), job control (3 items; eg, "I can work at my own pace"; α = 0.72), social support from supervisors (3 items; eg, "How reliable are your superiors when you are troubled?"; α = 0.81) and colleagues (3 items; eg, "How freely can you talk with your coworkers?"; α = 0.80), and workplace social capital (3 items; eg, "At our workplace, we have the attitude to work together"; α = 0.85) were measured using the Brief Job Stress Questionnaire (BJSQ).^27^ All items were scored on a 4‐point Likert scale (for job demands, job control, and workplace social capital: 1 = not at all to 4 = very much so; for social support: 1 = not at all to 4 = extremely). Higher scores mean higher job demands, job control, social support, and workplace social capital. The scores from each of the 3 items were averaged and used for analyses.

### 2.9 Positive and negative affect

Positive affect and negative affect were measured using 36 items (18 items for each) from previous Japan‐based studies. The scale was integrated using the Positive and Negative Affect Schedule (PANAS; eg, enthusiastic, attentive, proud, active, afraid, jittery, irritable, ashamed, and upset),^28^ which is a widely used mood measurement tool, and additional adjectives (eg, cheerful, in good spirits, extremely happy, nervous, restless or fidgety, and hopeless). These descriptors were added based on prior verification among the Japanese population. The past month was used as the time frame (ie, “During the past month, how much of the time did you feel…”). All items were scored on a 5‐point Likert‐type scale, ranging from 1 (never) to 5 (always). Positive and negative affect measure in this questionnaire was validated previously.^29^ The scores from each of the 18 items were averaged and used for analyses (Cronbach's α values: 0.95 [positive] and 0.92 [negative]).

### 2.10 Job and life satisfaction

Job and life satisfaction were also measured using questions from the BJSQ.^27^ BJSQ has been widely used for assessing stress factors and response and buffer factors in Japan. Job and life satisfaction measures consisted of 1 item each, "I am satisfied with my job" and "I am satisfied with my family life", respectively. They were scored on a 4‐point Likert scale (1 = dissatisfied to 4 = satisfied).

### 2.11 Eudaimonic well‐being at work

Eudaimonic well‐being at work was measured using the 24‐item University of Tokyo Occupational Mental Health well‐being scale (TOMH well‐being 24).^30^ This scale consists of eight factors, including role‐oriented future prospects, autonomy, role‐oriented positive perception, personal growth and development, negative schema, occupational self‐esteem, relationship, and meaningful work (each with 3 items). These items were scored on a 7‐point scale ranging from 0 (strongly disagree) to 6 (strongly agree). The reliability and validity of the TOMH well‐being 24 were confirmed in a previous study.^30^ The overall score (Cronbach's α = 0.95) and eight subscales were averaged and used for analyses.

### 2.12 Analysis

To test reliability, we calculated the Cronbach's α values, intra‐class correlation coefficients (ICCs), the standard error of measurement (SEM), and the smallest detectable change (SDC) values of the Japanese GAWS. Confirmatory factor analysis (CFA) and correlational analysis were performed to examine structural and convergent validity. We used SPSS Statistics 26.0 (SPSS Inc, Chicago, IL) and R version 4.0.0 for analyses.^31^

### 2.13 Internal consistency

To assess the internal consistency of the Japanese GAWS, Cronbach's α values were calculated for the total score and for each factor score (ie, SWE and MW). Per a previous study,^32^ a sample size of >100 was considered sufficient for methodological quality for the Cronbach's α level. Because a 2‐factor structure of the measure was confirmed in a previous study,^1^ we did not check the measure dimensionality but calculated Cronbach's α values for the total score and each factor score directly.

### 2.14 Test‐retest reliability

Longitudinal data were analyzed to assess test‐retest reliability. We excluded those who answered, "I moved to my workplace within the past two weeks" or "There was a big change in the workplace during the last two weeks." ICCs for total score and each factor score were calculated to investigate test‐retest reliability across the 2 weeks. We also calculated the SEM and SDC as standard measurement errors.^33^, ^34^ SEM represents the standard deviation of repeated measures within one participant, and SDC represents the smallest change one participant must exhibit on a measurement to ensure that the observed change is genuine and not just a measurement error.^33^ SEM was calculated as ([the standard deviation of all testing scores] × √[1 – ICC]^34^), and SDC was calculated as (1.96 × √[2 × SEM]^28^).

### 2.15 Structural validity

To confirm 2‐factor structural validity, CFA was performed among the 10 items using a robust maximum likelihood estimation in R. The original 2‐factor model (SWE [6 items] and MW [4 items] were explained by the two factors) and a 1‐factor model (all 10 items were explained by one factor) were assumed and tested in the following model fit indices: Chi‐square (χ2), comparative fit index (CFI), Tucker‐Lewis index (TLI), root mean square error of approximation (RMSEA), and standardized root mean square residual (SRMR). We considered the model a good fit on CFI and TLI >0.95 and RMSEA and SRMR <0.06.^35^ Per a previous study,^28^ the sample size required for factor analysis is at least five to seven times the item number, with a minimum of 100. Given that the Japanese GAWS has 10 items, an adequate number of participants (N = 100) were recruited.

### 2.16 Convergent validity

Pearson correlation coefficients (r) among the GAWS factors, (a) trait gratitude, (b) work‐related outcomes or factors, and (c) well‐being, were calculated to examine convergent validity. The minimum effect size for detection was 0.20 (ρ). Based on a sample size calculation using G*Power version 3.1.9.7,^36^ the necessary sample size was estimated to be >193 in the case of an α error probability of 0.05 and power (1 − β) of 0.80. Therefore, an adequate number of participants were recruited.

## 3 Results

### 3.1 Participant characteristics

**FIGURE 1:**
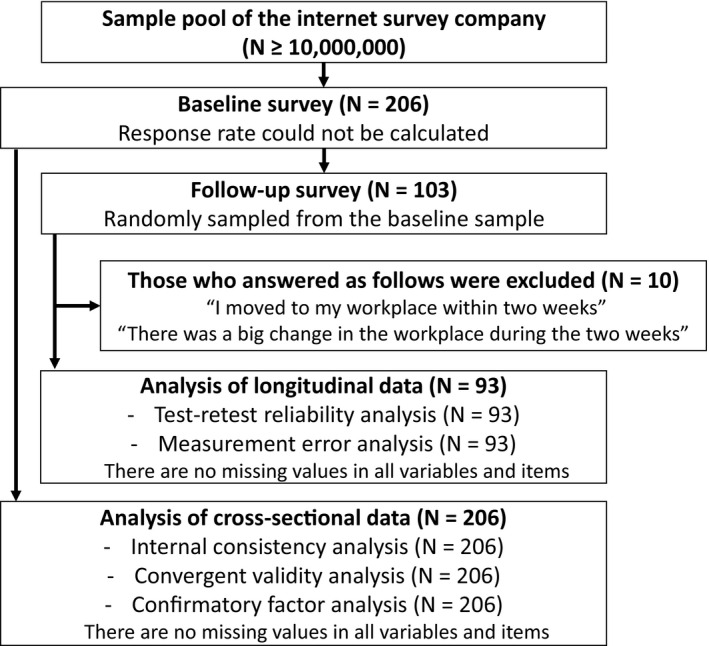
Participant flow chart

The participant flow chart is shown in Figure 1. The survey company stopped recruiting when the target number was reached; therefore, we could not determine the baseline response rate. In the 2‐week follow‐up survey, 103 workers were randomly sampled from the baseline pool to complete the questionnaire. For the Internet‐based survey, participants were required to answer all items, resulting in no missing values for any variables or items. Ten participants who answered, "I moved to my workplace within the past two weeks," or "There was a big change in the workplace during the last two weeks," at the follow‐up survey were excluded from the longitudinal analyses. Demographic characteristics of the participants at baseline and follow‐up are summarized in Table 1. On the baseline survey (N = 206, 132 men and 74 women, mean age = 42.2 ± 10.0 years), most participants had graduated from a university (50.0%) or had some college experience (19.9%). Most participants were married (66.5%), day‐time workers (87.4%), belonged to worksites with > 30 workers (37.4%), and engaged in occupations such as clerical (28.2%) or professional/technical jobs (20.9%) or service (11.2%). Characteristics of the participants on the follow‐up survey (N = 93, 63 men and 30 women, mean age = 43.8 ± 9.9) did not differ from those at the baseline.

**TABLE 1:** Demographic characteristics of the participants

	Baseline survey (N = 206)		Follow‐up survey (N = 93)	
	n (%)	Mean (SD)	n (%)	Mean (SD)
Gender				
Men	132 (64.1)		63 (67.7)	
Women	74 (35.9)		30 (32.3)	
Age		42.2 (10.0)		43.8 (9.9)
Educational status				
Elementary/junior high school	3 (1.5)		2 (2.2)	
High school	48 (23.3)		19 (20.4)	
College	41 (19.9)		22 (23.7)	
University	103 (50.0)		47 (50.5)	
Graduate school	11 (5.3)		3 (3.2)	
Marital status				
Married	137 (66.5)		65 (69.9)	
Not married	69 (33.5)		28 (30.1)	
Employment shift status				
Day shift	180 (87.4)		81 (87.1)	
Rotation/night shift	25 (12.2)		11 (11.9)	
Childcare leave	1 (0.5)		1 (1.1)	
Job category				
Clerical	58 (28.2)		24 (25.8)	
Profession/ technical job/ research job	43 (20.9)		16 (17.2)	
Service industry	23 (11.2)		10 (10.8)	
Sales job	22 (10.7)		12 (12.9)	
Production skilled job	17 (8.3)		10 (10.8)	
Others	43 (20.1)		21 (22.6)	
Size of workplace				
2‐5	38 (18.4)		23 (24.7)	
6‐9	35 (17.0)		10 (10.8)	
10‐19	35 (17.0)		12 (12.9)	
20‐29	21 (10.2)		13 (14.0)	
<30 employees	77 (37.4)		35 (37.6)	

### 3.2 Internal consistency and test‐retest reliability

Table 2 summarizes mean scores, Cronbach's alpha values (α), ICCs, SEMs, and SDCs for the GAWS factors. Cronbach's alpha coefficients ranged from 0.82 to 0.91. ICCs ranged from 0.81 to 0.90, indicating that approximately 80% of the variance in two time point measurements were explained by individuals. SDCs ranged from 0.74 to 1.02.

**TABLE 2:** Mean scores, internal consistency, and reliability of the Japanese version of the Gratitude at work scale (GAWS): N = 206

	Baseline Mean (SD)	Min‐Max	Cronbach's alpha	Follow‐up Mean (SD)^a^	Test‐retest Reliability (ICC)	SEM^a^	SDC^a^
Gratitude for a Supportive Work Environment (6 items)	3.02 (0.9)	1‐5	0.86	2.99 (0.8)	0.90^b^	0.28	0.76
Item 2	3.02 (1.2)	1‐5		2.81 (1.1)	0.87^b^	0.42	1.17
Item 4	3.00 (1.1)	1‐5		3.02 (1.1)	0.77^b^	0.52	1.43
Item 6	2.91 (1.1)	1‐5		2.97 (1.1)	0.76^b^	0.56	1.55
Item 8	2.87 (1.3)	1‐5		2.75 (1.2)	0.81^b^	0.55	1.51
Item 9	3.21 (1.1)	1‐5		3.31 (1.1)	0.74^b^	0.56	1.56
Item 10	3.11 (1.1)	1‐5		3.05 (1.0)	0.79^b^	0.50	1.38
Gratitude for Meaningful Work (4 items)	3.23 (0.8)	1‐5	0.82	3.06 (0.9)	0.81^b^	0.37	1.02
Item 1	3.46 (1.1)	1‐5		3.23 (1.0)	0.64^b^	0.64	1.77
Item 3	3.23 (1.0)	1‐5		3.05 (1.1)	0.72^b^	0.54	1.49
Item 5	3.23 (1.1)	1‐5		3.13 (1.0)	0.58^b^	0.69	1.92
Item 7	3.02 (1.1)	1‐5		2.83 (1.0)	0.83^b^	0.43	1.21
Overall GAWS (10 items)	3.11 (08)	1‐5	0.91	3.02 (0.8)	0.89^b^	0.27	0.74

^a^ N = 93. ICC: intra‐class correlation coefficient, SEM: standard error of measurement, SDC: smallest detectable change

^b^ *P* < .01

### 3.3 Structural validity

The results of CFA are listed in Table 3. Of the 1‐factor and 2‐factor models, the original 2‐factor hypothesized model demonstrated an acceptable fit (χ^2^ [34] = 67.58, CFI = 0.967, TLI = 0.956, RMSEA = 0.069, SRMR = 0.037). Standardized covariance among the two factors was 0.92, indicating strong correlations. Moreover, the 2‐factor model demonstrated a better fit than the 1‐factor model (Δχ^2^ [1] = 16.803, *P* < .001).

**TABLE 3:** Factor loadings of the Japanese version of the 10 GAWS items, factor correlations, and model fit in confirmatory factor analyses

	Factor loadings		Correlation coefficients in the 2‐factor model		
	1‐factor model	2‐factor model		SWE^b^	MW^b^
SWE Item 2	0.76^a^	0.77^a^	SWE	1.00	
SWE Item 4	0.66^a^	0.66^a^	MW	0.92^c^	1.00
SWE Item 6	0.88^a^	0.90^a^	Model fit	1‐factor	2‐factor
SWE Item 8	0.94^a^	0.96^a^	χ^2^ (*df*)	84.38 (35), *P* < .001	67.58 (34), *P* = .001
SWE Item 9	0.77^a^	0.79^a^	CFI	0.951	0.967
SWE Item 10	0.78^a^	0.79^a^	TLI	0.937	0.956
MW Item 1	0.53^a^	0.56^a^	RMSEA	0.083	0.069
MW Item 3	0.68^a^	0.71^a^	SRMR	0.042	0.037
MW Item 5	0.84^a^	0.88^a^	1‐factor model vs. 2‐factor model: Δχ2 (*df*)		16.803 (1), *P* < .001
MW Item 7	0.88^a^	0.89^a^			

Note

The robust maximum likelihood estimation method was used.

^a^ *P* < .05.

^b^ SWE, Supportive Work Environment; MW, Meaningful Work

^c^ *P* < .01

### 3.4 Convergent validity

Table 4 lists Pearson correlation coefficients (r) between the GAWS factors of (a) trait gratitude, (b) work‐related outcomes or factors, and (c) well‐being. The overall GAWS and the two factor scores had strong positive correlations with trait gratitude, job satisfaction, work engagement, and eudaimonic well‐being at work (0.52 ≤ r ≤ 0.68). Additionally, GAWS and the two factors had weak‐to‐moderate positive correlations with life satisfaction, positive affect, and organizational citizenship behavior (0.18 ≤ r ≤ 0.52). Moreover, the GAWS factors correlated slightly negatively with negative affect (−0.22 ≤ r ≤ −0.15). Among work‐related psychosocial factors, social support and social capital at work had moderate‐to‐strong correlations (0.28 ≤ r ≤ 0.67). Self‐reported work performance also had weak positive correlations with the GAWS factors (0.23 ≤ r ≤ 0.28).

**TABLE 4:** Convergent validity (r) of the Japanese version of the GAWS: N = 206

Variables	Mean (SD)	SWE	MW	Overall
GAWS ‐ Supportive Work Environment (SWE)	3.02 (0.9)	1.00	0.75^c^	0.96^c^
GAWS ‐ Meaningful Work (MW)	3.23 (0.8)	0.75^c^	1.00	0.90^c^
GAWS ‐ Overall	3.11 (0.8)	0.96^c^	0.90^c^	1.00
Trait Gratitude (GQ‐5)	4.67 (1.2)	0.54^c^	0.63^c^	0.61^c^
Work‐related outcomes/factors				
Work engagement – Overall (UWES)	2.31 (1.3)	0.64^b^	0.63^c^	0.68^c^
Vigor	2.22 (1.3)	0.60^c^	0.56^c^	0.62^c^
Dedication	2.52 (1.4)	0.64^c^	0.64^c^	0.68^c^
Absorption	2.19 (1.3)	0.59^c^	0.57^c^	0.62^c^
Organizational citizenship behavior (OCBS)	2.92 (0.7)	0.43^c^	0.49^c^	0.49^c^
Work performance (HPQ)	5.68 (2.0)	0.23^c^	0.28^c^	0.27^c^
Psychosocial factors at work (BJSQ)				
Job demands	2.67 (0.7)	‐0.07	0.01	‐0.01
Job control	2.64 (0.7)	0.40^c^	0.28^c^	0.38^c^
Social support from supervisors	2.37 (0.8)	0.65^c^	0.53^c^	0.64^c^
Social support from colleagues	2.54 (0.7)	0.55^c^	0.48^c^	0.56^c^
Workplace social capital	2.59 (0.7)	0.65^c^	0.59^c^	0.67^c^
Well‐being				
Positive affect^a^	2.64 (0.7)	0.49^c^	0.48^c^	0.52^c^
Negative affect^a^	2.43 (0.6)	‐0.22^c^	‐0.15^b^	‐0.20^c^
Job satisfaction (BJSQ)	2.46 (0.9)	0.59^c^	0.52^c^	0.60^c^
Life satisfaction (BJSQ)	2.78 (0.9)	0.18^b^	0.22^c^	0.21^c^
Eudemonic well‐being at work – Overall (TOMH well‐being 24)	3.03 (1.0)	0.57^c^	0.60^c^	0.62^c^
Role‐oriented future prospects	2.80 (1.3)	0.56^c^	0.61^c^	0.62^c^
Autonomy	2.94 (1.2)	0.26^c^	0.36^c^	0.32^c^
Role‐oriented positive perception	2.93 (1.4)	0.64^c^	0.64^c^	0.68^c^
Personal growth and development	3.26 (1.3)	0.54^c^	0.58^c^	0.59^c^
Negative schema	3.38 (1.1)	0.12	0.16^b^	0.15^b^
Occupational self‐esteem	2.86 (1.3)	0.40^c^	0.46^c^	0.45^c^
Relationship	3.08 (1.2)	0.61^c^	0.50^c^	0.60^c^
Meaningful work	3.00 (1.3)	0.48^c^	0.56^c^	0.55^c^

Note

GQ‐5: Gratitude questionnaire‐5, UWES: Utrecht work engagement scale, OCBS: Organizational citizenship behavior, HPQ: Health performance questionnaire, BJSQ: Brief job stress questionnaire, TOMH well‐being: The University of Tokyo Occupational Mental Health well‐being scale.

^a^ Positive affect and negative affect were measured by 36 items (18 items, respectively), to be more suitable for Japanese based on the previous study.

^b^ *P* < .05

^c^ *P* < .01

The exploratory examination showed comparatively weak correlations with job demands (−0.07 ≤ r ≤ 0.01) and a moderate positive correlation with job control (0.28 ≤ r ≤ 0.40).

## 4 Discussion

This study indicated that the Japanese GAWS demonstrated acceptable internal consistency, test‐retest reliability, and structural validity. It correlated highly with trait gratitude, work‐related outcomes or factors, and well‐being, suggesting good convergent validity. Overall, the Japanese GAWS proved to be a reliable and valid scale.

The scale showed good internal consistency of the total score and two subscale scores of gratitude for a supportive work environment and gratitude for meaningful work. The Cronbach's α values for the total and two subscales were 0.91, 0.86, and 0.82, respectively, which exceeded the stringent criterion of 0.80.^37^ The test‐retest reliability of these scales was acceptable, indicating that the scale scores were stable over the 2 weeks (ICC: 0.81‐0.90). These findings suggest that the Japanese GAWS is as reliable as the original version.^1^

Like the original version, in CFA, the 2‐factor model fits significantly better than the 1‐factor model. All indicators (CFI, TLI, and SRMR), except for RMSEA, had acceptable values, suggesting adequate structural validity. This indicates that among Japanese workers in an interdependent culture, there are two constructs of workplace gratitude: (a) supportive work environment and (b) meaningful work. Importantly, gratitude for meaningful work is defined slightly differently in Japan than in the West. During the cognitive debriefing, several workers expressed confusion with item 5, which focused on meaningful work ("your accomplishments at work?"), because they exclusively collaborate with others. Asians, including Japanese, may be more likely to feel workplace gratitude in a collective context rather than an individual context.

Criterion‐related and convergent validities were also well supported. As with the original GAWS, the correlation between the Japanese GAWS and trait gratitude suggested that the two concepts overlap but measure different aspects. The result was consistent with our hypothesis, indicating criterion‐related validity. Correlation between work‐related outcomes or factors was also consistent with the hypotheses. The results suggested that the GAWS was associated with important workplace outcomes and predictors. Work engagement, as a crucial variable associated with suicidal ideation or turnover intention,^24^, ^38^ is the key target outcome of workplace interventions. Regarding correlation with the three subscales, all of them showed large positive correlations as we assumed, and "dedication" had the strongest correlation. Dedication refers to being strongly involved in one's work and experiencing a sense of significance and pride.^39^ This is consistent with previous studies, showing that more grateful workers are highly motivated and are more likely to interpret situations positively.^3^, ^40^ Through a psychological mechanism of reciprocation,^17^ grateful workers may increase their dedication. Furthermore, psychosocial factors at work, such as job control, social support from supervisors and colleagues, and workplace social capital, have also shown a large correlation. According to the Job Demands‐Control‐Support model,^41^ these psychosocial factors play an important role in the workplace and predict a worker's well‐being and health.^14^ Seen from an organization‐level perspective, high levels of gratitude among workers can enhance the social support of others in the same workplace through organizational citizenship behaviors.^42^ The lack of correlation between Japanese GAWS and job demands is also consistent with the Job Demands‐Resources model.^14^ Job demands can have a negative effect on mental health but not on positive outcomes. The moderate correlation with work performance is also consistent with previous findings.^7^ In addition to the factors mentioned in previous studies (ie, mitigating factors [rancor and envy] that negatively affect performance), these psychosocial factors may also affect performance. Future research should examine the factors governing the associations of these meaningful variables with workplace gratitude.

The Japanese GAWS also correlated with indicators of well‐being. Interestingly, well‐being at work and job satisfaction were more strongly correlated than general well‐being outcomes, such as positive or negative affect and life satisfaction. Particularly strong correlations were found with eudaimonic well‐being at work (r = 0.62). Of the eight subscales, only "negative schema" showed a small correlation (0.12 ≤ r ≤ 0.16), contrary to the hypothesis. Negative schemas are defined as organized representations of an individual's past experiences that can influence current perceptions, thoughts, and behaviors.^43^ These are assumed to arise from adverse experiences early in life and remain latent.^44^ The results imply that having a negative schema and grateful feelings is mostly unassociated with each other. Since negative schemas are known to affect cognition and feelings,^44^ it is interesting that gratitude at work seemed to be independent of schemas.

Researchers and practitioners must determine whether changes in GAWS scores are practically meaningful. This study indicated a meaningful difference in total GAWS scores, ranging from 0.74 to 1.02 points, which can help evaluate a meaningful change in scale score when utilizing the scale in an intervention study or practice.

This study has limitations. First, response rate could not be calculated given the online survey administration, which may have caused a selection bias. For example, the participant pool may have been biased toward workers with higher happiness levels or lower stress levels. Furthermore, the Internet survey may have contained inappropriate answers. Second, the assessment of convergent validity may have included measurement error. Third, sensitivity was not examined, although it was examined in the original study.^1^ Fourth, we have not validated some items included in the COSMIN checklist (discriminant validity, cross‐cultural validity, and responsiveness). Fifth, the use of an Internet‐based survey limits the generalization.

In conclusion, the Japanese GAWS demonstrated good reliability and validity, showing a strong correlation with work engagement and eudaimonic well‐being. Future research and further intervention studies should explore factors related to workplace gratitude among workers.

## DISCLOSURE

*Approval of the research protocol*: The study protocol was approved by the research ethics committee of the Graduate School of Medicine and the Faculty of Medicine, The University of Tokyo, Japan (No. 2019216NI). *Informed Consent*: Informed consent was obtained from all participants according to the instructions given in the survey. The instructions explained that any identifying information would be removed from the data and assured protection of personal information. *Registry and the Registration No. of the study/trial*: N/A. *Animal Studies*: N/A. *Conflict of Interest*: Yu Komase, Kazuhiro Watanabe, and Natsu Sasaki declare no conflict of interest in connection with the paper. Norito Kawakami reports grants from Fujitsu LTD., SBAtWork Corp., personal fees from Occupational Health Foundation, Japan Dental Association, Sekisui Chemicals, Junpukai Health Care Center, Osaka Chamber of Commerce and Industry, and non‐financial support from Japan Productivity Center, outside the submitted work.

## AUTHOR CONTRIBUTIONS

YK, KW, NS and NK have made substantial contributions to the conception, design, and interpretation of data. Specifically, YK liaised with the original author, performed the translation of the scales from English to Japanese, cognitive debriefing, harmonization and integration of the measure, data collection, statistical analysis, and article writing. KW performed harmonization and integration of the measure, data collection, statistical analysis, and article writing. NS performed the translation of the scales from English to Japanese, harmonization and integration of the measure, data collection, and article writing. NK supervised the entire process.

## APPENDIX A 1. Gratitude at work scale

**Table:**

Instructions: Think about your experiences working at your current job. If you have more than one job think about the job at which you spend the most time. Then, indicate how often you feel grateful for the aspects of your job listed below. How often are you grateful for. . . . あなたが現在のお仕事で経験されていることを思い浮かべてください。複数のお仕事をされている場合は、最もたくさんの時間を費やすお仕事を思い浮かべてください。そして、その仕事に関する次のようなことに対して、あなたがどのくらいの頻度でありがたさを感じているかお答えください。						
Item	Question	Never 全くありがたさを感じない	Rarely あまりありがたさを感じない	Sometimes ときどきありがたさを感じる	Often しばしばありがたさを感じる	Almost Always いつもありがたさを感じる
1	Your interactions with those you serve (eg, your clients/customers/patients/students)? あなたが仕事やサービスを提供する人々とのつながりに対して (例: 顧客、取引先、患者、学生)	1	2	3	4	5
2	The salary and benefits you receive? あなたの給与や待遇に対して	1	2	3	4	5
3	The positive impact your job has on others? 自分の仕事を通じて、誰かの生活が良い方向に変わることに対して	1	2	3	4	5
4	The balance between your job and personal life? 仕事と私生活のバランスに対して	1	2	3	4	5
5	Your own accomplishments at work? 何かのおかげで自分が仕事を達成できたことに対して (例: 自分の能力、仕事の機会、周囲の環境)	1	2	3	4	5
6	The atmosphere/climate of your work environment? あなたの職場の雰囲気や文化に対して	1	2	3	4	5
7	Your ability to grow and learn from your job? 仕事を通して成長や学びが得られることに対して	1	2	3	4	5
8	The support you receive from your supervisor(s)? 上司からのサポートに対して	1	2	3	4	5
9	The support you receive from your coworker(s)? 同僚からのサポートに対して	1	2	3	4	5
10	Your autonomy in your workplace? 仕事の目標ややり方を自分で決めて働けることに対して	1	2	3	4	5
